# Supplementary material for: Causal Relationship between Aging and Anorexia Nervosa: A White-Matter-Microstructure-Mediated Mendelian Randomization Analysis
Source: Biomedicines. 2024 Aug 16;12(8):1874. doi: 10.3390/biomedicines12081874 (PMC11351342; doi:10.3390/biomedicines12081874)
Supplement: Supplementary file 1 [file biomedicines-12-01874-s001.zip › Supplementary Figure/Figure S4/Singe SNP plot of MR analysis for TL to ubm-b-2044.pdf]

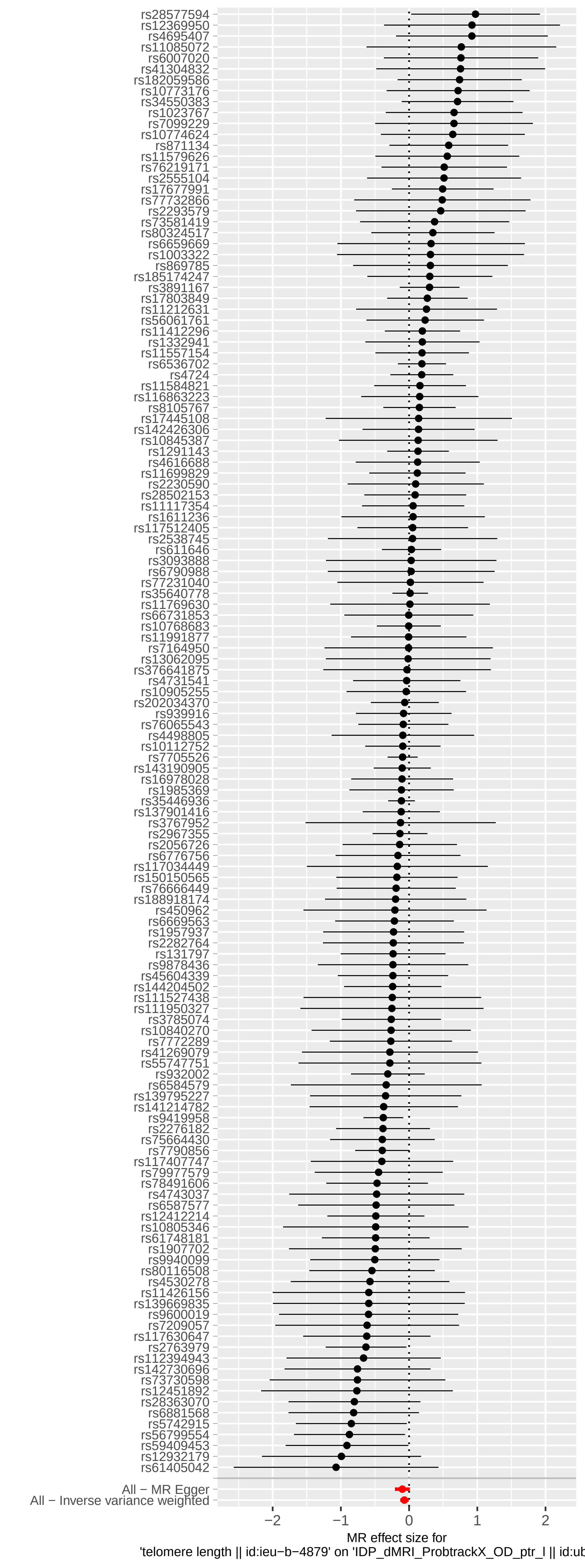
rs28577594
rs12369950
rs4695407
rs11085072
rs6007020
rs41304832
rs182059586
rs10773176
rs34550383
rs1023767
rs7099229
rs10774624
rs871134
rs11579626
rs76219171
rs2555104
rs17677991
rs77732866
rs2293579
rs73581419
rs80324517
rs6659669
rs1003322
rs869785
rs185174247
rs3891167
rs17803849
rs11212631
rs56061761
rs11412296
rs1332941
rs11557154
rs6536702
rs4724
rs11584821
rs116863223
rs8105767
rs17445108
rs142426306
rs10845387
rs1291143
rs4616688
rs11699829
rs2230590
rs28502153
rs11117354
rs1611236
rs117512405
rs2538745
rs611646
rs3093888
rs6790988
rs77231040
rs35640778
rs11769630
rs66731853
rs10768683
rs11991877
rs7164950
rs13062095
rs376641875
rs4731541
rs10905255
rs202034370
rs939916
rs76065543
rs4498805
rs10112752
rs7705526
rs143190905
rs16978028
rs1985369
rs35446936
rs137901416
rs3767952
rs2967355
rs2056726
rs6776756
rs117034449
rs150150565
rs76666449
rs188918174
rs450962
rs6669563
rs1957937
rs2282764
rs131797
rs9878436
rs45604339
rs144204502
rs111527438
rs111950327
rs3785074
rs10840270
rs7772289
rs41269079
rs55747751
rs932002
rs6584579
rs139795227
rs141214782
rs9419958
rs2276182
rs75664430
rs7790856
rs117407747
rs79977579
rs78491606
rs4743037
rs6587577
rs12412214
rs10805346
rs61748181
rs1907702
rs9940099
rs80116508
rs4530278
rs11426156
rs139669835
rs9600019
rs7209057
rs117630647
rs2763979
rs112394943
rs142730696
rs73730598
rs12451892
rs28363070
rs6881568
rs5742915
rs56799554
rs59409453
rs12932179
rs61405042
All − MR Egger
All − Inverse variance weighted
−2
−1
0
1
2
MR effect size for
'telomere length || id:ieu−b−4879' on 'IDP_dMRI_ProbtrackX_OD_ptr_l || id:ub
